# Genome-Wide Association Analysis across Endophenotypes in Alzheimer’s Disease: Main Effects and Disease Stage-Specific Interactions

**DOI:** 10.3390/genes14112010

**Published:** 2023-10-27

**Authors:** Thea J. Rosewood, Kwangsik Nho, Shannon L. Risacher, Sujuan Gao, Li Shen, Tatiana Foroud, Andrew J. Saykin, on behalf of the Alzheimer’s Disease Neuroimaging Initiative

**Affiliations:** 1 Indiana Alzheimer’s Disease Research Center, Indianapolis, IN 46202, USA; thearose@iu.edu (T.J.R.); srisache@iupui.edu (S.L.R.); sgao@iu.edu (S.G.); tforoud@iu.edu (T.F.); 2 Department of Medical and Molecular Genetics, Indiana University School of Medicine, Indianapolis, IN 46202, USA; 3 Department of Radiology and Imaging Sciences, Indiana University School of Medicine, Indianapolis, IN 46202, USA; 4 School of Informatics and Computing, Indiana University, Indianapolis, IN 46202, USA; 5 Department of Biostatistics, Indiana University School of Medicine, Indianapolis, IN 46202, USA; 6 Department of Biostatistics, Epidemiology and Informatics, The Perelman School of Medicine, Philadelphia, PA 19104, USA; li.shen@pennmedicine.upenn.edu

**Keywords:** genetics, GWAS, endophenotype, APOE, genetic interaction, cerebrospinal fluid biomarkers, magnetic resonance imaging, amyloid-PET, FDG-PET

## Abstract

The underlying genetic susceptibility for Alzheimer’s disease (AD) is not yet fully understood. The heterogeneous nature of the disease challenges genetic association studies. Endophenotype approaches can help to address this challenge by more direct interrogation of biological traits related to the disease. AD endophenotypes based on amyloid-β, tau, and neurodegeneration (A/T/N) biomarkers and cognitive performance were selected from the Alzheimer’s Disease Neuroimaging Initiative (ADNI) cohort (N = 1565). A genome-wide association study (GWAS) of quantitative phenotypes was performed using an SNP main effect and an SNP by Diagnosis interaction (SNP × DX) model to identify disease stage-specific genetic effects. Nine loci were identified as study-wide significant with one or more A/T/N endophenotypes in the main effect model, as well as additional findings significantly associated with cognitive measures. These nine loci include SNPs in or near the genes APOE, SRSF10, HLA-DQB1, XKR3, and KIAA1671. The SNP × DX model identified three study-wide significant genetic loci (BACH2, EP300, and PACRG-AS1) with a neuroprotective effect in later AD stage endophenotypes. An endophenotype approach identified novel genetic associations and provided insight into the molecular mechanisms underlying the genetic associations that may otherwise be missed using conventional case-control study designs.

## 1. Introduction

The complex nature of Alzheimer’s disease (AD) is clear from multiple studies that have identified genetic risk factors and potential contributors to the disease, but the underlying drivers of disease are not well understood. The known genetic risk factors still do not fully explain the genetic heritability of AD. There is increasing evidence that late onset AD is largely determined by multiple, small effects and low penetrance genetic factors [1,2]. Identifying novel genetic effects that influence AD risk is important for enhancing the understanding of the pathogenesis of the disease. Alternative approaches are needed to further identify the missing genetic contributors to susceptivity and progression of AD and to facilitate the path toward genetically informed therapeutic approaches.

The National Institute on Aging and Alzheimer’s Association (NIA-AA) established a research framework for defining AD by its biomarkers rather than its clinical consequences. This framework categorizes the biomarkers of AD into three primary groups: β amyloid deposition (A), pathologic tau (T), and neurodegeneration (N) [3]. Biomarker endophenotypes for AD have been used to increase statistical detection power and can provide valuable insight into the molecular mechanisms underlying AD [4]. An ideal endophenotype lies downstream of genetics risk and upstream of the “observable” phenotype, in this case, cognitive changes in AD. The A/T/N framework represents the pathophysiology underlying the disease, providing quantifiable measures of the pathology of the disease for genetic association. 

The Alzheimer’s Disease Neuroimaging Initiative (ADNI) cohort provides a wide variety of biomarkers highly associated with AD, and several studies have utilized this dataset for genetic analysis [5]. The ADNI also provides an opportunity to evaluate multiple endophenotypes together in a shared dataset, allowing a comprehensive analysis of genetic associations with AD endophenotypes.

In this study, two models were used to evaluate genetic associations with AD endophenotypes: A main effect model of overall genetic associations with baseline measures of endophenotypes and an SNP by Diagnosis (SNP × DX) interaction model of genetic associations within diagnostic stages of AD. The main effect model follows a traditional linear regression approach applied to the individual biomarkers and identifies overall genetic effects on endophenotypic measures across all subjects and diagnostic classification groups. Cognitive measures were included in this analysis as a quantitative marker for clinical outcomes. Primary consideration is given to SNPs that meet significance in biomarker endophenotypes, particularly those that meet significance in multiple endophenotypes.

The SNP × DX model analyzes the relation between genetic variation and endophenotype in the context of stage-specific clinical diagnostic classification: cognitively normal older adults (CN), early mild cognitive impairment (EMCI), late MCI (LMCI), and AD. Progression along the stages of the clinical syndrome of AD varies across patients, with evidence that genetic factors influence the heterogeneity of this disease [6,7]. Previous studies in the ADNI cohort have suggested an interaction with specific known AD genes and diagnostic groups, but a robust genome-wide analysis across multiple phenotypes has not been performed [8]. The main effect model is well suited for identifying genetic effects that influence endophenotypes across all subjects and diagnostic groups, providing a contrast between individuals that are cognitively normal and those that are on the AD spectrum of diagnosis. However, genetic contributors that influence stage-specific pathophysiological changes may not be detected by this approach. Including an interaction term between genetic markers and diagnostic group is hypothesized to facilitate identification of stage-specific genetic effects. Here, we present analysis of the ADNI endophenotype data by a model including both SNP mail effects and SNPxDX interactions.

## 2. Materials and Methods

### 2.1. Study Participants

Participants from the Alzheimer’s Disease Neuroimaging Initiative Phase 1 (ADNI-1) and the next two extensions (ADNI-GO/2) [9] were included in this study and were obtained from the Alzheimer’s Disease Neuroimaging Initiative (ADNI) database (adni.loni.usc.edu (accessed on 10 September 2023)). The ADNI was launched in 2003 as a public-private partnership, led by Principal Investigator Michael W. Weiner, MD. The primary goal of ADNI has been to test whether serial magnetic resonance imaging (MRI), positron emission tomography (PET), other biological markers, and clinical and neuropsychological assessment can be combined to measure the progression of mild cognitive impairment (MCI) and early Alzheimer’s disease (AD). For up-to-date information, see www.adni-info.org (accessed on 10 September 2023). Further information about these studies, participant enrollment, protocols, and other information can be found at www.adni-info.org. Written informed consent was obtained from each participant, and all protocols were approved by each site’s Institutional Review Board.

### 2.2. Genotyping and Imputation

Whole blood samples from the ADNI participants were genotyped on the Illumina Human 610-Quad BeadChip, the Illumina HumanOmniExpress Beadchip, or the Illumina Omni 2.5 M platform (Illumina, Inc., San Diego, CA, USA). After standard quality control (QC) procedures of GWAS data for samples and SNPs [10], genotype imputation and calling was performed over each data set separately using the Haplotype Reference Consortium Panel r1.1. *APOE* genotypes for rs429358 and rs7412, described as *APOE* ε2/ε3/ε4 status, were genotyped separately, as described previously [8]. Due to data limitations, to avoid population stratification confounding from underrepresented populations present in the dataset, non-Hispanic ADNI participants of European ancestry (N = 1565) were selected for this analysis by genetic clustering using HapMap 3 genotype data and multidimensional scaling (MDS) analysis. We note that this restricted analysis was performed to reduce the well-known influence of population stratification effects on genetic associations [11,12]. Separate large-scale efforts are underway to address the differential genetic architecture of AD in multi-ethnic populations, and the latest phase of ADNI is explicitly focusing on racial/ethnic diversity.

### 2.3. Selected Phenotypes

Biomarkers were selected based on previous studies for association with AD pathology [13]. Measures of 17 phenotypes at baseline were selected to represent the key A/T/N biomarker groups of AD, and an additional Cognitive performance (C) category. Amyloid-β (A) biomarkers are represented by one region of interest (ROI) measured from [18F]Florbetapir amyloid PET scans and CSF amyloid-β 1-42 peptide (Aβ1-42), Tau (T) biomarkers by CSF total tau (t-tau) and phosphorylated tau (p-tau), Neurodegeneration (N) biomarkers measured from MRI scans (8 ROIs) and FDG PET scans (3 ROIs), and Cognitive performance (C) as composite scores developed by Crane et al. [14] for memory (MEM) and executive functioning (EF). Selection of MRI and FDG ROIs was based on previous studies of AD pathology and progression and to cross-sectionally represent the disease across stages [15,16]. Table 1 presents the full list of phenotypes and sample sizes.

### 2.4. Genetic Association Analysis

Genome-wide association analysis for the main effect was performed separately for each phenotype in PLINK v1.9 [17]. Association was evaluated across 5,406,480 genotyped and imputed variants for each phenotype. All phenotypes were adjusted for age, sex, and the first two principal components of the genetic population by inclusion in the linear model. MRI and cognitive measures were additionally adjusted for education, and MRI measures were adjusted for intracranial volume.

The analysis was performed with and without the *APOE* e2/e3/e4 genotype as a covariate to account for the effects of the *APOE* allele on genetic effects. To fully account for an *APOE* genotypic effect, the *APOE* genotype was coded as dummy variables indicating 1 = e2e2, 2 = e2e3, 3 = e3e3, 4 = e2e4, 5 = e3e4, and 6 = e4e4. 

MRI field strength was identified as an additional covariate for MRI phenotypes; however, MRI field strength was directly tied to the ADNI phase with ADNI Phase 1 participants and ADNI GO/2 participants receiving 1.5 Tesla and 3 Tesla MRI, respectively. No significant effect from the ADNI phase was identified in non-MRI measures. MRI phenotypes were adjusted for age, sex, education, ICV, and MRI field strength by using a regression model based on the cognitive normal group. The resulting β coefficient of the MRI field strength was then used to adjust the MRI phenotype across all subjects using the following formula:

Field Strength-Adjusted MRI Phenotype = MRI Phenotype − (β_MRI Field Strength_ × (MRI Field Strength − Mean CN MRI Field Strength))

The field strength-Adjusted MRI phenotype variable was then treated the same as the remaining phenotypes and run in the linear model with the remaining selected covariates.

Principal component analysis of the 17 selected phenotypes identified 6 principal components, explaining 85% of the variance across phenotypes. An adjusted study-wide significance threshold, based on the conventional 5 × 10^−8^, was set at *p* ≤ 8.33 × 10^−9^ (5 × 10^−8^ divided by 6 components). The conventional genome-wide threshold of *p* ≤ 5 × 10^−8^ and a suggestive association threshold *p* ≤ 1 × 10^−5^ were noted for the purpose of comparison across phenotypes and for additional analyses.

To identify the peak SNPs for a genetic region, SNPs meeting at least the suggestive association threshold were trimmed based on Linkage Disequilibrium (LD) analysis. SNPs were sorted by *p*-value, and those in LD with R^2^ greater than 0.2 were considered to be in the same gene region for the purposes of identifying top SNPs for a genetic region.

A separate analysis was performed adjusting for diagnosis to remove the main effect of diagnosis, identifying SNP-phenotype associations not specific to AD. Diagnosis was categorized as CN, EMCI, LMCI, and AD.

### 2.5. SNP × DX Interaction Analysis

The SNP × DX interaction analysis was performed through linear regression computed in R, with each phenotype as the dependent variable and SNP, diagnosis, and the interaction term (SNP × DX) as independent variables. The same covariates as above were applied (Table 1). DX in the interaction term was coded as an ordinal logistic variable, interpreted in four groups: CN, EMCI, LMCI, and AD. Ordinal coding interpreted these groups as having linear relationships between them with unknown spacing (CN < EMCI < LMCI < AD). The same thresholds and trimming methods as the main effect analysis above were applied on the interaction term of the model to determine significant interaction effects.

### 2.6. Functional Analysis

Variants were annotated for variant position and nearest gene using ANNOVAR (Version 2017-07-17). Study-wide significant results were submitted to the Genotype-Tissue Expression (GTEx) Portal to determine *cis* expression quantitative trait locus (eQTL) effects. RegulomeDB [18] was referenced for potential regulatory effects for SNPs of interest.

ADNI microarray gene expression profiling from blood samples and post-mortem brain tissue RNA sequencing data from the Accelerating Medicines Partnership Alzheimer’s Disease (AMP-AD) consortium were used for expression quantitative trait loci (eQTL) analysis. The top SNPs from the results of this study were evaluated within these datasets using the MatrixEQTL 2.1.0 R package [19] to identify potential functional expression effects in blood and across different brain regions.

## 3. Results

### 3.1. Genome-Wide Association Results

**Figure 1:**
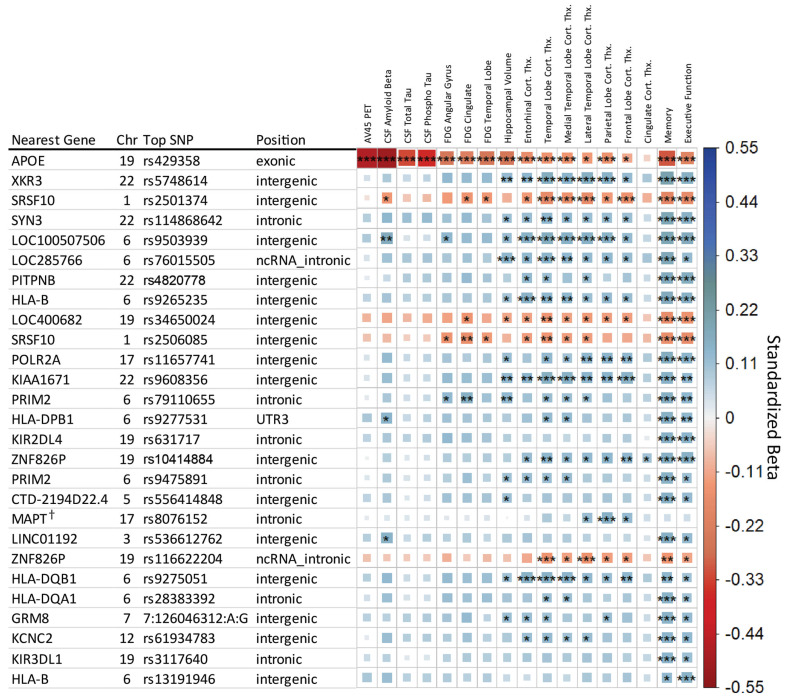
Matrix of main effect analysis results. Each row indicates the top SNP for a genetic region after LD trimming, and each column represents an AD endophenotype. Ordered based on minimum *p*-value across the row. The asterisks represent the *p*-value of the association with [***] indicating meeting the study-wide significant threshold (*p* ≤ 8.33 × 10^−9^), [**] the conventional genome-wide threshold (*p* ≤ 5 × 10^−8^), and [*] the suggestive association threshold (*p* ≤ 1 × 10^−5^). The color and box size relate to the β value effect size for a given association, with larger box size relating to distance from zero in either positive (blue, suggesting neuroprotective) or negative (red, suggesting neuropathological effect) direction. ^†^ SNP retains significance when including DX as a covariate.

After LD trimming, a total of 27 genetic regions were identified as having study-wide significance with at least one endophenotype. Figure 1 provides an overview of these top SNPs and their associations with each endophenotype. The majority of findings are associated with composite scores for memory or executive functioning. Of primary interest are the nine SNPs that met study-wide significance in the A/T/N endophenotypes in addition to the cognitive scores. Many associations showed effect directions suggesting a neuroprotective effect, with four findings apart from *APOE* showing a risk direction in association with the minor allele: rs2501374 and rs2506085 in an intergenic region on chromosome 1, rs9503939 intergenic on chromosome 19, and rs116622204 intronic to pseudogene *ZNF826P*.

Including the *APOE* e2/e3/e4 allele as a covariate reduced the 27 study-wide significant genetic regions to 16. Six SNPs maintained study-wide significance in a non-cognitive measure, rs5748614 (near *XKR3*), rs2501374 (near *SRSF10*), rs9503939 (near LOC100507506), rs9608356 (near *KIAA1671*), rs8076152 (within *MAPT*), and rs116622204 (within *ZNF826P*).

Including diagnosis as a covariate identified only one SNP that retained study-wide significance, rs8076152, which is intronic to *MAPT*, associated with parietal lobe cortical thickness (MRI). A suggestive association was also found in frontal and lateral temporal lobe cortical thicknesses (MRI).

### 3.2. Known AD-Associated Genetic Regions

Association analysis identified rs429358 (*APOE* e4 allele) on chromosome 19 as strongly associated with 13 of the 17 endophenotypes, with suggestive influence on the others. *APOE* e4 was most strongly associated with measures of amyloid-β, followed by measures of tau, glucose metabolism, and overall cognitive memory score. Strong associations were observed for surrounding SNPs in LD with *APOE* e4 in and near *TOMM40*, *APOC1*, and *NECTIN2* genes on chromosome 19.

Associations were also observed for regions in or near *HLA-DQA1, HLA-DPA1*, and *HLA-DRB1*, which have been identified in large-scale AD case/control studies [20].

### 3.3. SNPxDiagnosis Results

**Figure 2:**
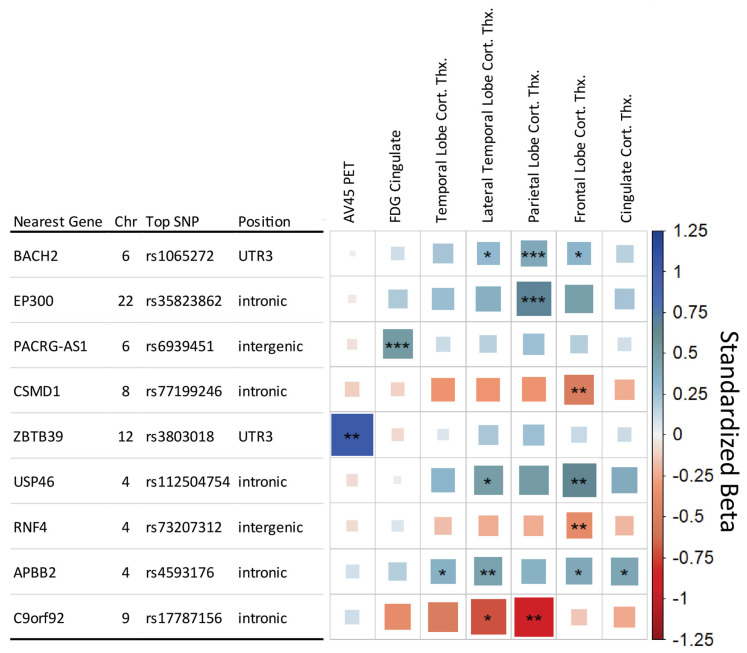
Matrix of SNP x Diagnosis analysis results. Each row indicates the top SNP for a genetic region after LD trimming, and each column represents an AD endophenotype. Endophenotypes showing no level of significance were removed for clarity. The asterisks represent the *p*-value of the association with [***] indicating meeting the study-wide significant threshold (*p* ≤ 8.33 × 10^−9^), [**] the conventional genome-wide threshold (*p* ≤ 5 × 10^−8^), and [*] the suggestive threshold (*p* ≤ 1 × 10^−5^). The color and box size relate to the β value effect size for a given association, with larger box size relating to distance from zero in either positive (blue, suggesting neuroprotective) or negative (red, suggesting neuropathological effect) direction.

**Figure 3:**
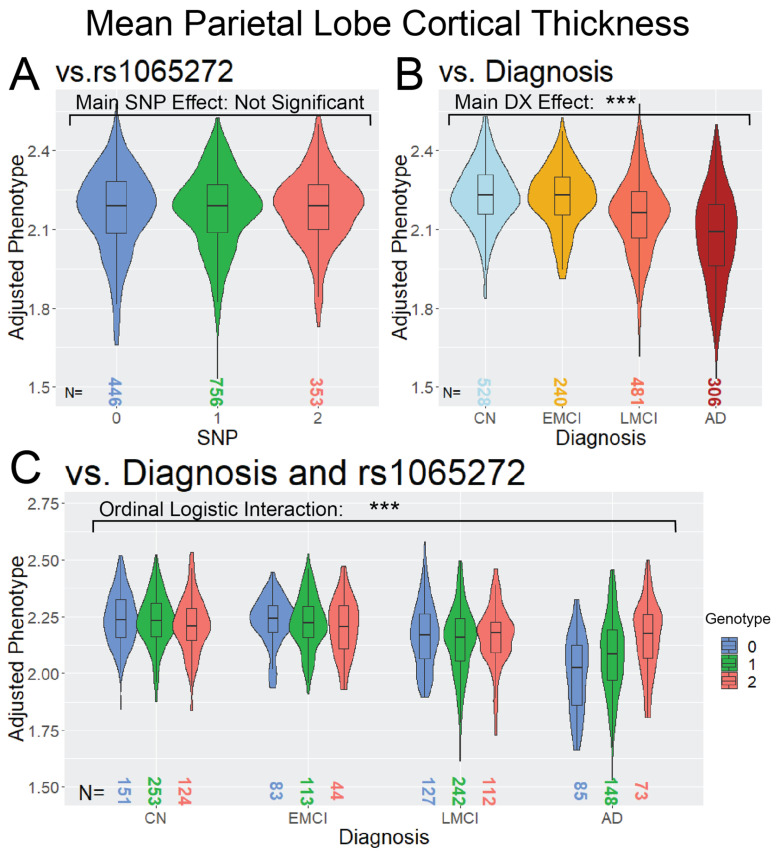
Violin and boxplot distribution of Parietal Lobe Cortical Thickness, stratified by (**A**) rs1065272 SNP, (**B**) Diagnosis, and (**C**) SNP and Diagnosis. (**A**) represents the main effect analysis, (**B**) the association with Diagnosis, and (**C**) the SNP × DX interaction association, with DX codes as an ordinal logistic variable of distinct categories with known ordinal relation (CN < EMCI < LMCI < AD). The asterisks represent the *p*-value of the association with [***] indicating meeting the study-wide significant threshold (*p* ≤ 8.33 × 10^−9^).

The SNP × DX model identified three study-wide significant interaction SNPs (Figure 2). The first two were primarily associated with parietal lobe cortical thickness, rs1065272 located in the 3′ untranslated region of *BACH2* on Chromosome 6 and rs35823862 intronic to *EP300* on Chromosome 22. The *BACH2* SNP also showed suggestive associations with the Frontal Lobe and Lateral Temporal Lobe cortical thickness measures. A study-wide significant SNP was also associated with FDG PET in the cingulate and is located in an intergenic region on Chromosome 6 near *PACRG-AS1*. The three SNPs showed a neuroprotective effect in later stages of AD, as represented in Figure 3 with rs1065272 in *BACH2*.

The additional six genetic regions met the less strict conventional genome-wide significant threshold (*p* ≤ 5 × 10^−8^) (Figure 2). Including *APOE* genotype as a covariate in the SNP × DX model identified the same SNPs as without *APOE* adjustment.

### 3.4. Functional Results

The RegulomeDB analysis identified a score of 2b for the SNP rs2501374, indicating that there is evidence for transcription factor binding with a DNase footprint and DNase peak but no matched transcription factor. Other SNPs did not show suitable evidence in the RegulomeDB.

In the GTEx database, ADNI whole blood, and AMP-AD eQTL analysis, nine main effect SNPs were identified as being associated with altered expression. For the SNP × DX analysis, two SNPs were associated with altered expression. Table 2 provides a summary of eQTL findings between these approaches.

## 4. Discussion

Systematic analysis of genetic associations with key AD endophenotypes identified nine significant genetic regions, including regions previously associated with AD as well as several novel genetic associations. Analysis with a stage-specific approach further identified genetic effects that modulate endophenotypes in later stages of AD. The significant genes and their relation to AD from the main effect and SNP × DX approaches are summarized in Table 3. The differences in genetic association detected by the two models highlight the need for thoughtful consideration and assessment of analytic models for AD as a complex disease. The variants detected are among many that have been identified as contributing to AD risk. Understanding how, where, and when these variants are affecting the disease through models such as those used in this study will be important for an enhanced understanding of the disease and developing genetically informed therapeutic approaches.

The SNPs identified in the main effect approach are largely in intergenic or uncharacterized regions, making functional analysis more challenging. The intergenic SNP rs2501374 proved particularly robust in this analysis. There is evidence of transcription factor binding in this region, though no direct eQTL evidence is available for this SNP within evaluated datasets. The nearest gene, *SRSF10*, is an alternative splicing gene which has general implications with AD pathogenesis [21,22]. *SRSF10* has known effects in enhanced lipogenesis [23] and affects alternative splicing of IL1RAP [24], which has been associated with AD pathology [25]. An SNP in the same region that might be separate in relation to linkage disequilibrium, rs2506085, shows an eQTL effect on *RCAN3*. This gene has been previously shown to be differentially expressed in AD [26]. The *RCAN* family, particularly *RCAN1*, has been shown to be associated with the inverse link between AD and cancer [27] as well as related to mitochondrial disruption in AD [28]. Another robust SNP is near *XKR3*, which belongs to a family of phospholipid scramblases which have potential implications in apoptotic signaling [29], with eQTL evidence supporting a functional effect on expression in whole blood samples.

The HLA region has proven to be of interest in AD, particularly the HLA Class II region [20,30,31,32], with evidence for the HLA Class I regions as well [33,34]. Five independent HLA genetic regions were identified as having study-wide significance in at least one endophenotype, with additional HLA Class I related markers in *KIR3DL1* and *KIR2DL4*. eQTL analysis identified an association of these HLA region SNPs with HLA gene expression levels. However, study-wide significance is reduced in the HLA-related SNPs when adjusting for *APOE* allele status, with only rs9265235 near *HLA-B* and rs9277531 near *HLA-DPB1* maintaining study-wide significance in cognitive measures. Further study in how HLA-related genes associate and interact with AD endophenotype pathology is warranted.

SNPs with study-wide significance in a cognitive measure but suggestive association in other endophenotypes may provide insight as well. An SNP near *POL2RA* (rs11657741) shows a significant eQTL effect on *CHRNB1*, an acetylcholine receptor protein. There is evidence to suggest a role of the cholinergic system in AD [35]. *PRIM2* plays a role in DNA replication through Okazaki fragment formation, with DNA replication stress playing a potential role in AD pathology [36]. The SNP rs11486842 intronic to *SYN3* shows evidence of a small eQTL effect on *TIMP3*, which has been shown to regulate *APP* processing and *APOE* receptor proteolysis [37]. *SYN3* itself is involved in neurotransmitter release and synaptogenesis, and has been shown to be downregulated in hippocampal CA1 neurons in a tauopathy mouse model [38].

An SNP intronic to *MAPT* (rs8076152) is of potential interest, as it resides within the tau protein gene and is associated with neuroprotective effects in the parietal lobe. Evidence from this study suggests that the SNP is not directly related to AD as it retains its association with MRI measures when diagnosis is included as a covariate. While ADNI is an AD-focused cohort, this effect may be related to other forms of dementia present within the dataset. *MAPT* is an important gene in a variety of tauopathies [39,40].

The main effect analysis showed many significant associations with the composite cognitive measures. This is in part due to the cognitive scores having the highest N, resulting in better statistical detection power for association. MRI measures were close in sample size and corroborated many of the cognitive associations. Cognitive measures differ from classic endophenotypes but act as surrogate quantitative measures for dimensions driving diagnostic decisions, and therefore represent the downstream effects of other biological marker-based phenotypic changes. Based on these results, utilizing a quantitative trait representative of diagnosis rather than a binary case/control status indicator could enhance the detection of genetic association in AD. However, caution is needed in interpreting the cognitive measures alone without other endophenotypes, as those measures may be confounded by non-AD factors.

Many of the findings within this study indicate a neuroprotective effect direction in relation to the minor allele, that is, they are associated with a healthier measure of the associated phenotype such as less atrophy in MRI measures. While often the genetic risk effects, or neuropathological effect direction in this study, are of more interest due to their contribution to disease risk, in the context of this study, these SNPs may provide insight into factors that contribute to resistance and resilience to pathological change [41,42]. Both protective and risk effects can aid in understanding what biological pathways are involved in the course of the disease, and what mechanisms might be targetable for therapeutic development. These loci may be preferentially engaged in certain aspects of the pathology compared to others, and thus influence the heterogeneity of disease progression [43,44]. There may also be regional implications for cerebral pathology. For example, the risk SNP near *SRSF10* shows little association effect size for hippocampal volume measures, yet very strong association effects with temporal lobe cortical measures. 

Due to sample size differences, this dataset is best suited for evaluating MRI and cognitive phenotypes and lacks some power in PET and CSF measures for proper comparison across measures. However, the observed effect sizes can provide insight into shared association as well as patterns of effect magnitude across endophenotypes. Where sample size is lacking power to yield statistical significance, the effect sizes demonstrate patterns across endophenotypes. Despite sample size differences, SNPs rs2501374 and rs950939 showed suggestive association in CSF amyloid-β levels, consistent with study-wide significance seen in those SNPs in MRI and cognitive measures.

Outside of *APOE* and its surrounding regions, there was very little significance seen in Amyloid-β and Tau biomarkers. This is in part due to the lower sample size of these measures, but other factors may contribute. Amyloid deposition in cognitively normal individuals [45] may affect detection power of AD-specific genetic effects, and more complex models to account for these subjects may be needed. Additionally, polygenic risk score studies have provided evidence of common AD genetic markers having little contribution toward risk of amyloid deposition, distinct from *APOE*, which contributes greatly toward amyloid deposition [46].

The analysis performed here for main effect of SNP does not consistently replicate the top genetic regions identified in large-scale AD GWAS studies [20,47], outside of *APOE* and genes in the HLA class II region. The main difference is approach, with the large-scale GWAS utilizing a case/control analysis where this study utilized an endophenotype approach. Despite the relatively smaller sample size, the endophenotype approach may provide power for identifying genetic effects specific to selective aspects of AD-related pathology. These genetic effects relate directly to AD mechanisms or AD-relevant biology (Table 3) that contribute to differences in disease pathology, where case/control targets more generalized risk. In a highly heterogeneous disease like AD, a generalized approach may not capture genetic effects specific to biology that will be important in developing a more personalized medicine approach. 

The SNP × DX approach provides an alternative assessment of genetic effects occurring in specific stages of AD, with study-wide significant findings with AD-specific implications. The top SNP in the *BACH2* gene region is located in its 3′ UTR, suggesting possible post-transcriptional regulatory effects of the SNP. *BACH2* is a transcriptional regulator involved in processes like NF-κB signaling, apoptosis in response to oxidative stress, nuclear import of actin, and CD4(+) T-cell differentiation. It has also been shown to be upregulated in β-amyloid-treated SH-SY5Y neuroblastoma cells [48]. The top genetic association in *EP300* has some evidence of the eQTL effect (Table 2) and is one of many strongly associated SNPs that blanket the *EP300* gene. *EP300* has been shown to be strongly associated as a candidate “Master Regulator” in AD genetic network analysis [49], and the p300 protein has been implicated in general neurodegeneration through its epigenetic mechanisms [50] and promoting tau secretion and propagation [51]. Many of the findings through this approach are significant in the parietal region. The parietal region is typically affected later in AD disease progression [52]. Though this interaction approach might be better powered for detecting changes in the final stages of disease, more complex modeling would be necessary for detecting intermediate stage changes.

**Table 3:** Summary of significant genetic findings. ● indicates SNPs within the gene were significant in the indicated study. “-” indicates a novel finding within this study.

Gene Region	Cross-Sectional	SNP × DX	Previous Link to AD	Potential Biological Relevance in AD
*APOE*	●		Major AD Risk locus; Multiple GWAS	Lipid Transporter involved in CNS Maintenance
*XKR3*	●		-	Apoptotic signaling
*SRSF10*	●		-	Alternative splicing; enhanced lipogenesis
*RCAN3*	●		Differentially expressed in AD [26]	Immune, T Cell development
*HLA-B*	●		Multiple GWAS [33,34]	Immune
*KIAA1671*	●		AD Multi-Omic Weighted GWAS [53]	Immune
*HLA-DQB1*	●		Multiple GWAS [20,30,31,32]	Immune
*HLA-DQA1*	●		Multiple GWAS [20,30,31,32]	Immune
*ZNF826P*	●		-	Unknown
*BACH2*		●	Upregulated in β-amyloid-treated SH-SY5Y neuroblastoma cells [48]	Apoptosis; Nuclear import of actin; Transcriptional regulator of NF-κB
*EP300*		●	Associated with altered bile acids in AD; shown to be involved in AD regulatory network [49]	Mediates cAMP gene regulation
*PACRG-AS1*		●	-	Lewy bodies; Heat shock, apoptosis

SNPs that did not meet the study-wide significant threshold in the SNP × DX results but met the less strict conventional genome-wide significant threshold are nonetheless still of heuristic value and biological interest. The SNP intronic to the gene Amyloid β Precursor Protein Binding Family B Member 2 (*APBB2*), which has been previously associated with AD [54], was identified as being associated with larger volumes in the lateral temporal lobe in later AD stages. Three SNPs are of note for being associated with a neurodegenerative direction. Two show effect in the frontal lobe, and one shows a strong effect in the parietal lobe within the RNA gene C9orf92. SNP × DX findings such as these can provide insight into how different genetic mechanisms modify disease progression in AD.

As with many GWAS studies, these findings are limited by the selected population and sample sizes, and further replication in independent larger cohorts is warranted. Larger sample sizes would benefit both models, and with the SNP × DX approach, it would allow more sophisticated modeling of interactions in earlier stages of the disease that could not be interpreted with confidence given the current sample size limitation. Future studies will include gene and pathway enrichment, functional analysis in multi-omic datasets, and application of endophenotypic associations in a polygenic risk score model.

In summary, our findings show that an endophenotype approach can identify novel genetic associations with links to AD as well as provide insight into the identified associations. Endophenotypes allow for more complex models, such as the SNP × DX approach used here, which may be necessary in identifying genetic effects that contribute to AD risk and progression that might otherwise be missed in conventional models. The findings identified here may provide insight into potential genetically informed therapeutic targets or provide insight relevant for enhanced assessment of genetic risk. Expanding on the techniques utilized in this study through more comprehensive modeling and larger samples will likely provide further power for new discoveries.

## Figures and Tables

**Table 1:** List of selected endophenotypes, their N, and covariates applied during regression analysis indicated by •. * Analysis performed with and without *APOE* covariate. ^†^ Values were pre-adjusted for MRI-Field strength where applicable.

Selected Endophenotype	N	Genetic PC1	Genetic PC2	APOE E2/E3/E4 Allele *	Age	Sex	Education	Intracranial Volume	MRI Field Strength ^†^
Memory Composite Score	1565	•	•	•	•	•	•		
Executive Function Composite Score	1565	•	•	•	•	•	•		
Bilateral mean hippocampus volume	1555	•	•	•	•	•	•	•	•
Bilateral mean entorhinal cortex thickness	1555	•	•	•	•	•	•	•	•
Bilateral mean frontal lobe thickness	1555	•	•	•	•	•	•	•	•
Bilateral mean cingulate thickness	1555	•	•	•	•	•	•	•	•
Bilateral mean parietal lobe thickness	1555	•	•	•	•	•	•	•	•
Bilateral mean temp. lobe thickness	1555	•	•	•	•	•	•	•	•
Bilateral mean medial temp. lobe thickness	1555	•	•	•	•	•	•	•	•
Bilateral mean lateral temp. lobe thickness	1555	•	•	•	•	•	•	•	•
Mean FDG PET SUVR in Angular Gyrus	1158	•	•	•	•	•			
Mean FDG PET SUVR in Cingulate	1158	•	•	•	•	•			
Mean FDG PET SUVR in Bilateral Mean Temp. Lobe	1158	•	•	•	•	•			
[18F]Florbetapir amyloid PET	791	•	•	•	•	•			
CSF amyloid-β 1-42 peptide	981	•	•	•	•	•			
CSF Total Tau	1103	•	•	•	•	•			
CSF Phosphorylated Tau	1103	•	•	•	•	•			

**Table 2:** Summary of eQTL findings in ADNI whole blood analysis, GTEx database lookup, and AMP-AD brain eQTL. ● indicates significant eQTL association with the SNP in the indicated brain region. “NA” indicates there was no association with the gene in the dataset, “-“ indicates the SNP was not available in the dataset. An * indicates the SNP is from the SNP × DX association analysis. WB = Whole blood, Brain-Hipp = Brain Hippocampus, Brain-cb = Brain-Cerebellum.

SNP	eQTL Gene	ADNI Blood eQTL FDR *p*-Value	GTEx Tissue	AMP-AD FDR Significant					
				MSBB				Mayo	
				BM-10	BM-22	BM-36	BM-44	Cerebellum	Temporal Cortex
rs2506085	RCAN3	NA	NA						●
rs2506085	FUCA1	NA	Whole Blood						
rs28383392	HLA-DQA1	3.15 × 10^−93^	-	●	●	●		●	●
rs28383392	HLA-DQB1	9.15 × 10^−92^	-	●	●	●	●	●	●
rs3117640	FCAR	1.38 × 10^−3^	-						
rs3117640	KIR2DS4	7.93 × 10^−19^	-						
rs3117640	KIR3DL1	6.53 × 10^−4^	-						
rs3803018 *	RDH16	NA	Brain-cb						
rs3803018 *	STAT6	3.92 × 10^−4^	NA						
rs5748614	TPTEP1	NA	Whole Blood						
rs5748614	XKR3	2.96 × 10^−14^	Whole Blood						
rs587750081	ZNF826P	1.19 × 10^−18^	-						
rs631717	KIR2DS4	7.26 × 10^−29^	Whole Blood						
rs631717	KIR3DL1	8.78 × 10^−12^	Whole Blood						
rs631717	KIR3DL2	3.68 × 10^−6^	Whole Blood						
rs8076152	KANSL1-AS1	NA	Whole Blood/Brain-Hipp	●			●	●	●
rs8076152	KANSL1-AS1	NA	Brain-cb	●	●		●	●	●
rs8076152	LRRC37A4P	8.40 × 10^−20^	Whole Blood/Brain-Hipp					●	●
rs8076152	ARL17B	NA	Brain-cb					●	●
rs8076152	MAPK8IP1	2.48 × 10^−7^	Whole Blood/Brain-Hipp						
rs9265235	HLA-B	9.87 × 10^−6^	NA		●		●		
rs9265235	C4A	NA	Brain-Hipp	●					
rs9275051	HLA-DQA1	1.23 × 10^−50^	Whole Blood	●	●	●	●	●	●
rs9275051	HLA-DQA2	NA	Whole Blood/Brain-Hipp						
rs9275051	HLA-DQB2	NA	Whole Blood/Brain-Hipp						
rs9275051	HLA-DQB1	7.66 × 10^−45^	Whole Blood/Brain-Hipp	●	●	●	●	●	
rs9275051	HLA-DRB1	NA	Brain-cb	●	●			●	●
rs35823862 *	ADSL	NA	NA						●
rs35823862 *	MCHR1	NA	Brain-cb						●

## Data Availability

Data for this study were obtained from the Alzheimer’s Disease Neuroimaging Initiative. Access to this data can be obtained by visiting https://adni.loni.usc.edu/data-samples/access-data/ (accessed on 10 September 2023).
